# The neonatal Fc receptor in cancer FcRn in cancer

**DOI:** 10.1002/cam4.3067

**Published:** 2020-05-05

**Authors:** Diana Cadena Castaneda, Guillaume Brachet, Caroline Goupille, Lobna Ouldamer, Valérie Gouilleux‐Gruart

**Affiliations:** ^1^ Université de Tours Tours France; ^2^ CHRU de Tours Tours France; ^3^ Université de Tours INSERM Tours France

**Keywords:** antigen presentation, cancer, cancer prognosis, cross‐presentation, FCGRT, immune complexes, immunosurveillance, Neonatal Fc receptor, tumor metabolism

## Abstract

Since the neonatal IgG Fc receptor (FcRn) was discovered, it was found to be involved in immunoglobulin recycling and biodistribution, immune complexes routing, antigen presentation, humoral immune response, and cancer immunosurveillance. The latest data show that FcRn plays a part in cancer pathophysiology. In various types of cancers, such as lung and colorectal cancer, FcRn has been described as an early marker for prognosis. Dysregulation of FcRn expression by cancer cells allows them to increase their metabolism, and this process could be exploited for passive targeting of cytotoxic drugs. However, the roles of this receptor depend on whether the studied cell population is the tumor tissue or the infiltrating cells, bringing forward the need for further studies.

## Graphical abstract

The neonatal Fc Receptor has long been considered only for its biodistribution and recycling roles. Recently, new functions and hopes emerged from the latest discoveries, especially in the context of cancer immunosurveillance. These new functions and related hopes for cancer treatment are reviewed here.

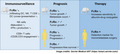

**Highlights**

- Novel immune and pathophysiological functions of FcRn are reviewed
- FcRn plays a role in NK cell maturation
- FcRn could become part of an early prognosis biomarker score
- Inside cancer cells, FCGRT downregulation leads to increased tumor growth
- In FcRn‐expressing tumors, FcRn is described in micrometastase detection

## 1 THE NEONATAL Fc RECEPTOR: A NEVER‐ENDING STORY

The neonatal IgG‐Fc receptor (FcRn) is a heterodimeric protein, whose existence was hypothesized way before its formal biochemical identification.^1^, ^2^ Brambell, who was the first to suggest its existence, initially thought that IgG and albumin salvage and recycling could each depend on distinct mechanisms, which is not the case. Indeed, albumin and IgGs both bind with FcRn, although on two different binding sites.^3^ This heterodimeric intracellular receptor is composed of an α‐chain encoded by the *FCGRT* gene, noncovalently bound to the β2‐microglobulin. Serum IgGs and albumin undergo continuous fluid phase pinocytosis. They are directed to early endosomes, where the acidic environment favors histidine protonation on an interface that encompasses the CH2 and CH3 domains of the IgG and the α1 and β subunits of FcRn.^4^ This electrostatic interaction dramatically increases the affinity of IgGs for FcRn, and they bind in a 2:1 fashion, two FcRn molecules flanking monomeric IgGs.^5^ IgGs, as albumin, are then either recycled back to the membrane or transcytosed to the other side of the cell, and released in the extracellular media upon interface deprotonation, in response to neutral ambient pH. This long‐known and well‐characterized recycling role has quantitative consequences on IgG serum levels, making FcRn a central element of IgG homeostasis.^4^, ^6^, ^7^ In spite of its name, the “neonatal” Fc receptor is permanently expressed throughout life, and has a wide expression profile across tissues and cell types.^8^, ^9^ However, the precise cell contingent quantitatively responsible for IgG homeostasis is yet to be identified. The latest research suggests that macrophages could play an important role quantitatively.^10^

Recently, FcRn‐related immune functions in humoral immune response and cancer immunosurveillance raised the interest of the scientific community. During the last decade, FcRn was notably shown to take part in antigen phagocytosis and immune complex (IC) direct‐ or cross‐presentation.^11^, ^12^, ^13^ Knockout experiments on MDCK2 cells showed that the intracellular sorting of IgGs and ICs involves complex pathways and multiple intracellular trafficking routes.^14^ In addition to these in vitro experiments, in vivo studies showed that FcRn was able to transport albumin‐based ICs across the epithelial barrier into the *lamina propria* and deliver them to dendritic cells (DCs) in mice.^15^

Fc gamma receptors (FcγRs) also participate in the routing of IgG‐based ICs. As a consequence, efficient humoral response against IgG1‐based ICs even occur in FcRn‐KO mice.^16^ FcγRs are a family of immunoglobulin domain containing receptors, which can either be activating or inhibitory, high‐ or medium‐to‐low‐affinity and transmembrane or lipid‐anchored.^17^ The engagement of these receptors, differentially expressed on various specialized cell contingents, affects the nature of the immune response toward immune complexes.^18^ A recent review presents the available data describing the roles of the various FcγRs in IC routing together with FcRn.^11^ Besides Fcγ receptors, the classical complement pathway initiator C1q was also shown to be involved in a cooperation with FcRn for IC routing.^19^ These functions have been extensively reviewed in comprehensive papers from renowned specialists and will not be further described here.^11^, ^20^, ^21^

## 2 MODULATION OF FcRn EXPRESSION AND CONSEQUENCES ON IMMUNOSURVEILLANCE

Recent literature focusing on FcRn in cancer pathophysiology shows that its role exceeds these canonical functions. The involvement of FcRn in cancer immunosurveillance through cross‐presentation was first described by Baker et al.^22^ Tumor‐specific ICs were cross‐presented in an FcRn‐dependent manner, and CD8^+^ T‐cell‐mediated tumor clearance relied on the presence of tumor‐specific antibodies.^22^, ^23^ In a murine model of spontaneous colorectal cancer, homozygous inactivation of *FCGRT* led to increased tumor progression and metastasis.^22^ In this work, DCs from *FCGRT* ^−/−^ mice exhibited decreased IL‐12 synthesis capacity. As a consequence, infiltrating CD8^+^ T‐cells were less abundant and less activated, with an impaired response upon CD3 and CD28 engagement. In a murine lung cancer model, the author showed the importance of FcRn engagement for CD8^+^ CD11b‐ DC priming, using IgG mutants that either had enhanced or inhibited binding to the receptor. In summary, FcRn downregulation in infiltrating immune cells could be responsible for defective immune responses.

The latest literature shows that FcRn deficiency could also impair NK cell maturation and differentiation.^24^ Such abnormalities were observed in *FCGRT*‐deficient mice, their NK cells being inefficient at IFN‐γ synthesis upon chemical or cytokine stimulation. The abundance and effectiveness of NK cells inside the tumor microenvironment have been associated with better prognosis in several human cancers including lung, bladder, thyroid, and prostate.^25^, ^26^, ^27^, ^28^ Therefore, if FcRn expression were to be modulated throughout oncogenic processes, for example, through a crosstalk between cancer stem cells and tumor infiltrating cells, it could hamper cancer immunosurveillance by decreasing cross‐presentation and thus antibody‐ and cell‐dependent‐cytotoxicity.

## 3 FcRn AS A POTENTIAL PROGNOSTIC AND DIAGNOSTIC BIOMARKER

In breast cancer, was FcRn found to be expressed in both the epithelial cells of mammary glands and axillary lymph node metastases. In this study, the neoplastic change did not modify the expression of the receptor.^29^ Indeed, the authors notice that the metastatic cells detected in the draining lymph node still express FcRn. They suggest that a co‐staining of FcRn and cytokeratin in the intraoperatory biopsies could help detect and remove micrometastases.^29^ An undisclosed number of biopsies were dissected by the authors, concluding that FcRn is generally expressed in breast carcinomas. On the other hand, *FCGRT* downregulation has been documented in progressive breast cancer types, and the expression levels seem to depend on the disease stage and aggressiveness.^30^ The quantitation of FcRn expression levels as a biomarker for prognostic purposes has been patented a decade ago, even though their use in routine practice has not been established so far.^31^ In hepatocellular carcinoma biopsies, overall downregulation of *FCGRT* has also been correlated with poor prognosis.^32^ In nonsmall‐cell lung cancer, similar conclusions were drawn for the quantitative analysis of *FCGRT* mRNA expression. A total of eighty biopsies were studied, and FcRn downregulation could be correlated with poor prognosis.^33^ In this context, specific quantitation of *FCGRT* mRNA levels proved that patients with preserved FcRn expression had significantly higher progression‐free survival than the patients with low expression. This parameter was shown to have a prognostic value, even at early stage, being significantly correlated with subsequent outcome. Thus *FCGRT* mRNA downregulation in both cancer and noncancer cells in nonsmall‐cell lung cancer can be associated with weaker antitumor response and shorter progression‐free survival. The expression of the *FCGRT* gene is often studied in primary tumor material, but precise information about which cell types express it or not is often missing. Indeed, the scientific added value of quantifying the expression of the *FCGRT* gene separately between cancer‐ and noncancer cells is only as recent as the aforementioned literature involving FcRn in tumor immunosurveillance. Thus, FcRn could be used as a biomarker for prognosis in several types of cancer.

## 4 *FCGRT* GENE REGULATION AND INTERACTION

Ongoing studies focus on *FCGRT* gene regulation in various tissues in humans, and several factors involved in its epigenetic control in human lung tissue were recently described.^34^, ^35^ Notably, the results so far indicate that specific microRNAs (miRNAs) and DNA methylation control the expression of *FCGRT*, and that this gene is differentially expressed between cancer cells and adjacent tissues. These results could maybe reveal deeper immune disorders underlying tumor pathophysiology in some cases. This hypothesis is supported by another finding from Cejas et al.^35^ In their work, regulator miRNA also altered hepatic *FCGRT* expression, with a potential impact on serum levels of both IgGs and albumin.

Artificial intelligence‐based approaches also corroborate this pivotal role of FcRn in tumorigenesis. An algorithm was set to analyze available data regarding multiple gene expression without a priori*,* making it a powerful tool to gain insight into tumorigenesis‐involved genes.^36^ This in silico study, based on artificial neural network inference, revealed that FcRn behaved as a central hub between several correlated genes in Ewing's sarcoma. The authors concluded that *FCGRT* played a role in connecting genes associated with the *EWSR1/FLI1* fusion gene, which encodes a well‐known powerful transcription activator involved in the pathophysiology of this disease.^37^ Even though this methodology does not give any mechanistic information, it strongly encourages further research on the topic, especially because of the wide distribution of FcRn across tissues, unlike that of FcγRs, poorly documented outside of immune cells.

## 5 FcRn DYSREGULATION SUSTAINS TUMOR DEVELOPMENT

FcRn dysregulation has been described in numerous and various cancer types and cell lines.^29^, ^33^, ^38^, ^39^ Downregulation could be associated with increased tumor growth and poor prognosis. But the mechanism by which downregulation promotes tumor growth long remained unclear. Amongst 11 commercially available cancer cell lines, FcRn expression at the protein level was shown to be low or undetectable in nine, including breast, prostate, and lung cancer.^39^ Keeping in mind that FcRn both binds albumin and IgGs, its underexpression leads to an abolishment of albumin salvage from for the lysosomal digestion. Thus, the pinocytosed albumin is catabolized instead of being recycled. The subsequent raise in intracellular glutamate levels increases the cell metabolism, enhancing tumor cell growth in a SCID murine model.^39^ These results need to be confirmed in immunocompetent mice and in humans. Indeed, hypoalbuminemia is known to be associated with shorter progression‐free survival and worse outcome in several malignancies, such as colorectal cancer, ovarian cancer, renal cell carcinoma, biliary tract cancer, and palliative cancer care.^40^, ^41^, ^42^, ^43^, ^44^ Thus, it would be helpful to understand whether the hypoalbuminemia is caused by cancer cells themselves or not, which would give another perspective on administrating fractionated albumin. Paradoxically, it seems that FcRn overexpression also increases tumor fitness in Balb/cAnRj‐Foxn1^nu/nu^ mice, compared to FcRn knockout tumors.^38^ The proposed mechanism relies on the nutrient cargo role of albumin. More than a source of aminoacids for the cancer cells, it behaves as a transporter for important substrates such as fatty acids and thyroxin. Both up and downregulation of FcRn could sustain tumor development. The papers describing these dysregulations at least agree on this: further experiments are to be undertaken to clarify the role of FcRn in cancer cell pathophysiology.

FcRn‐related albumin metabolism could bring forward new treatment perspectives and help stratify patients, based on FcRn expression levels. Indeed, an increase in albumin uptake and high metabolic turnover constitutes a strong argument for the design of albumin‐based drug conjugates. FcRn‐expressing cells would recycle the albumin‐drug conjugate, whereas cancer cells with a high metabolic intake due to FcRn underexpression would catabolize it and release the cytotoxic drug. Relative tissue selectivity would be provided by the high albumin uptake and lysosomal degradation, limiting off‐target toxicity. Existing biopharmaceuticals such as Nab‐paclitaxel should therefore be assessed for the passive targeting of FcRn‐deficient tumors. Nab‐paclitaxel is a paclitaxel‐conjugated albumin‐nanoparticle system, which was developed to increase the bioavailability and tolerability of paclitaxel. This treatment happens to be used in patients suffering from metastatic breast cancer.^45^ A high amount triple‐negative tumors underexpress *FCGRT,* explaining the high and selective accumulation of nab‐paclitaxel, and encouraging the use of similar molecules in other types of aggressive solid tumors. Nude mice grafted with pancreatic ductal adenocarcinoma cells were recently and successfully treated with an albumin‐doxorubicin conjugate, providing a rationale for the design of such passively targeted drugs.^46^ In contrast, in FcRn overexpressing tumors, albumin‐based drug products could only function if releasing of the cytotoxic moiety occured before the endosome.

## 6 PIVOTAL ROLE FOR FcRn IN CANCER PATHOPHYSIOLOGY AND MEDICAL IMPLICATIONS

**FIGURE 1:**
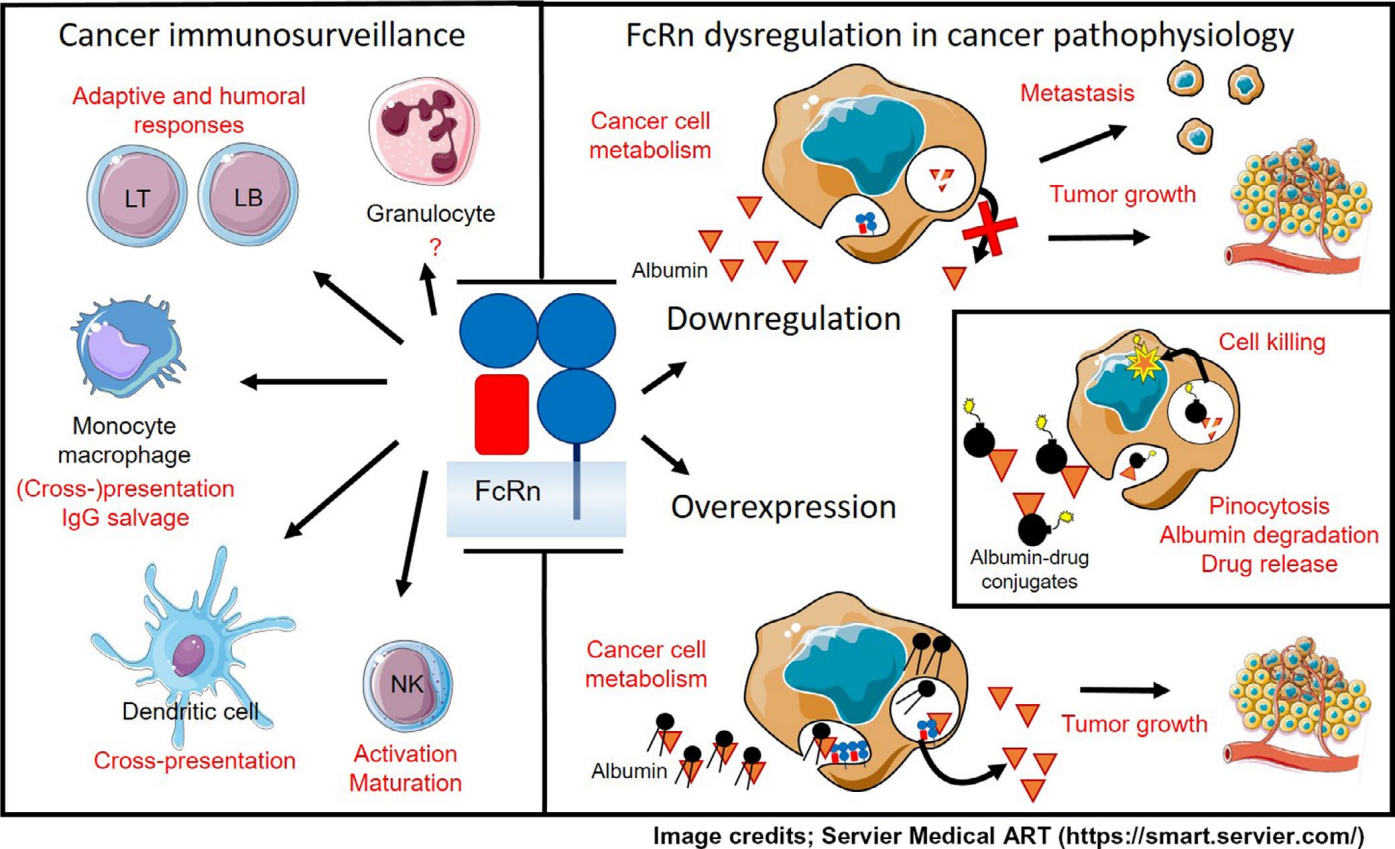
Documented mechanistic insights into the role of the neonatal Fc receptor in cancer immunosurveillance (left panel) and the effects of its dysregulation on tumor growth and metastasis (right), either in the case of overexpression (bottom) or underexpression (top). The inset at the right side describes the expected mechanism of action of albumin‐drug conjugates on cancer cells that underexpress FcRn. Albumin is pinocytosed, and directed to endosomes. In the absence of the neonatal Fc receptor, it is digested instead of being recycled back to the membrane. Treatment with albumin‐drug conjugates leads to the catabolism of the protein moiety and cellular accumulation of the cytotoxic drug. LB and LT stand for B‐ and T‐lymphocytes respectively. Albumin is represented as orange triangles, cytotoxic drug moiety is depicted as bombs, and nutrients as a ball with two side chains, also in black

In summary, as described in the Table 1 and Figure 1, new functions for FcRn have been described during the last decade. Despite the growing interest for this receptor, little is known about the regulation of *FCGRT*, especially during carcinogenesis. The underexpression of this receptor is associated with decreased immunosurveillance. In cancer cells, FcRn dysregulation promotes tumor growth, by mechanisms that still need to be clarified. Depending on this, albumin‐based drug conjugates could be used for the treatment of tumors that dysregulate FcRn. Finally, the available literature suggests a pivotal role of *FcRn*, encouraging mechanistic research around its involvement in cancer pathophysiology.

**TABLE 1:** FcRn in cancer pathophysiology: summary of the findings in the literature and methodology

Affirmation	Methodology	Publication	References
Macrophages account for most IgG recycling quantitatively	In vivo, murine model	Challa DK et al, in mAbs, 2019	[8]
Intracellular sorting of IgGs and ICs involves complex pathways and multiple intracellular trafficking	In vitro, cell‐based assay	Nelms B, et al, in J Cell Biol. 2017	[14]
FcRn‐KO mice are able to mount an efficient humoral response against IgG1‐based ICs	In vivo, murine model	Arnoult C, et al, in J Immunol 2017	[15]
*FCGRT* deletion leads to increased tumor progression and metastasis in mice with spontaneous colorectal cancer	In vivo, murine model	Baker K, et al, in Immunity. 2013	[21]
FcRn‐dependent cross‐presentation of tumor‐specific‐IgG‐based immune complexes is necessary to elicit CD8^+^ T‐cell‐mediated tumor clearance	In vivo, murine model	van Montfoort N, et al, in Eur J Immunol. 2012	[22]
FcRn is involved in NK cell maturation and differentiation	In vivo, murine model	Castaneda DC, et al, in Front Immunol. 2018	[23]
FcRn is expressed in both the epithelial cells of mammary glands and axillary lymph node metastases	Human biopsies	Cianga P, et al, in Hum Immunol. 2003	[29]
*FCGRT* downregulation has been documented in progressive breast cancer types and could be correlated to aggressiveness	Human biopsies	Jansen MPHM, et al, in J Clin Oncol. 2005	[30]
In hepatocellular carcinoma downregulation of *FCGRT* has also been correlated with poor prognosis	Human biopsies	Shi L, et al, in BMC Cancer. 2016	[32]
In biopsies from nonsmall‐cell lung cancer patients, *FCGRT* mRNA was under expressed, and downregulation was correlated with poor prognosis	Human biopsies	Dalloneau E, et al, in Oncotarget. 2016	[33]
Specific microRNAs (miRNAs) and DNA methylation control the expression of *FCGRT*	In vitro*,* cell‐based assay	Ferguson DC, et al, in Pharm Res. 2018	[34]
*FCGRT* epigenetic control by DNA methylation in the myocardium and liver	In vitro*,* cell‐based assay Human biopsies	Cejas RB, et al, in Sci Rep. 2019	[35]
An artificial intelligence analysis of gene expression in a database without a priori	In silico database analysis	Tong DL, et al, in PloS One. 2014	[36]
FcRn low or undetectable breast, prostate and lung cancer cells use albumin as an amino acid source, which promotes tumor growth	In vitro cell line analysis*,* In vivo, xenografted mice	Swiercz R, et al, in Oncotarget. 2017	[38]
Successful treatment of apancreatic ductal adenocarcinoma model albumin‐doxorubicin conjugate	In vivo, murine model	Liu H, et al, in J Control Release, 2019	[45]
hFcRn overexpression significantly increases cancer cell growth, and is seen in 8/10 tested human cancer tissue types	In vitro cell line analysis*,* In vivo, xenografted mice	Larsen MT, et al In	[46]

## COMPETING INTERESTS

The authors have no conflicts of interest to disclose.

## AUTHOR CONTRIBUTIONS

DCC VGG and GB wrote the paper; DCC, GB, CG, LO, and VGG reviewed the paper and take responsibility for the final version; VGG supervised the writing, LO and CG provided clinical information; GB edited and submitted the manuscript.

## DECLARATION STATEMENTS

Ethics approval and consent to participate: Not applicable.

Consent for publication: All authors have seen and approve the final version of this manuscript.

Availability of data and material: The data presented in this manuscript are available on request.
